# The medical experience of a patient with a rare disease and her family

**DOI:** 10.1186/s13023-016-0401-7

**Published:** 2016-02-29

**Authors:** Roberta Garau

**Affiliations:** University of Aberdeen, Aberdeen, UK

**Keywords:** Patient experience, rare cancer

## Abstract

This letter considers the main challenges that people with rare diseases and their families face: delay in diagnosis, lack of appropriate support and information, and impaired access to treatment. The differences in medical experience between a patient with a rare disease and one with a common one are made through the use of a real-life example: the diagnosis of leiomyosarcoma received by my mother. I highlight how patients with rare disease are often misdiagnoses and how their symptoms are often overlooked. I also highlight the isolation patients with rare diseases and their families experience due to the lack of knowledge about their condition, the struggle to access treatment and the small amount of information and evidence based medicine for managing rare conditions.

This article was the winning entry in the Findacure essay contest ‘The Student Voice’. More information about Findacure can be found at http://www.findacure.org.uk.

## Correspondence

*“Unfortunately it is cancer. It is called undifferentiated pleomorphic uterine sarcoma, but the doctors are not sure, it could be a leiomyosarcoma. They have never seen a similar case before.”*

I remember the day my father called me to give me the terrible news. I could not believe that it was my own mother affected by a disease with such a frightening name. Remembering the spelling so I could Google it was hard enough. I was only at the end of my first year of medical school and thought it was normal that I had never heard of it. I soon realised that it was too rare to be covered in lectures. In the few hours after my father’s call I discovered that uterine sarcomas account for 1 % of uterine cancers [1], and uterine cancer in turn makes up only 2.5 % of cancer diagnoses [2]. I could not find a specific incidence rate for her cancer, because no one was ever sure of the diagnosis. Suddenly, I realised that the challenges my family was going to face to receive appropriate care were greater than the ones we would have faced had this cancer been more common.

A rare disease is defined by the European Union as a “disease that affects less than 5 in 10,000 people” [3]. There are between 6000 and 8000 rare diseases [3]. Collectively, they are common: in the UK 1 in 17 people will be affected by a rare disease at some point in their life [3]. Rare diseases are heterogeneous: they can result from a single gene mutation, often inherited, or from a combination of environmental and genetic factors.

In this essay I consider the main challenges that people with rare diseases and their families face: delays in diagnosis, lack of appropriate support and information, and impaired access to treatment.

*“I have to send you to A&E. This bleeding is not normal, it could be a warning sign of something serious.”*

My mother was lucky in the sense that her cancer presented with a common alarm symptom: heavy post-menopausal bleeding. The GP recognised that the symptoms could be serious and she was immediately referred to secondary care. In a week she had a diagnosis and a plan to access appropriate treatment. Many people with rare diseases present with “odd” symptoms and experience long delays before the correct diagnosis is made. A survey from Rare Diseases UK from 2010 reports that only 26 % of respondents had a diagnosis in less than 3 months, 46 % had to wait more than one year [4]. Some rare diseases are similar to common ones, causing multiple misdiagnoses. An alarming 30 % of patients who answered the survey received three or more misdiagnoses. The survey reports the story of Jo Gray, a woman affected by multiple endocrine neoplasia type 2B (MEN-2B) [4]. She had to wait three years before receiving a diagnosis of MEN-2B. Her mother and son were subsequently found to also have the condition. The delay in diagnosis put her, and her loved ones, at serious risk of complications such as heart failure, stroke and even death [4]. She presented to her GP with retching, vomiting, severe headache attacks, palpitations and breathlessness [4]. She was a new mother and was misdiagnosed with postnatal depression [4]. MEN-2 syndromes are extremely rare conditions, affecting only around 1000 families worldwide [5]. It is understandable that the first differential diagnosis in her GP’s list was not a MEN syndrome; postnatal depression is a common diagnosis and probably a correct one for most new mothers with similar symptoms. However, the inability to listen to Mrs. Gray’s concerns, to see her desperation, to acknowledge the failure of treatments for her diagnosis was inexcusable. These shortcomings are a threat to a positive medical experience for rare and common diseases alike. In both cases, there is a risk of forgetting the person behind the symptoms. The focus should never move from the patients’ experience to the doctor’s idea of how a disease should progress and how a patient should behave to fit in.

*“I have never done an operation for this kind of tumour. The specialist is in Milan, he would be technically more proficient but it could be a long wait. You have to decide what to do.”*

On the broad spectrum of cancer patients, my mother was fortunate in that her tumour was at least operable. However, her treatment journey is a microcosm of the battle people with rare diseases face. The gynaecologist in my city was brutally honest in stating that he had no experience carrying out the required operation. The best person to do so was 430 miles away in Milan, and we would have to wait a month before he became available. The other option was to opt for immediate surgery locally. But how does one weigh up the value of a surgeon with experience of a rare cancer against the risk of giving a rapidly proliferating cancer an extra month to grow? I will never know if we made the right decision by opting to wait and travel to Milan. If we had chosen to act immediately perhaps my mother’s metastases would have come earlier, later, or not at all. Unfortunately that is the world in which sufferers of rare diseases and their families must live - a world of hypotheticals. No evidence exists to direct patient or clinician down a particular path.

My entire family was desperate to know everything they could about my mother’s cancer to make the best decisions. Unfortunately, all we were given by way of an explanation at the local hospital was that it was a very rare, aggressive disease. The internet became the main source of information: websites targeted at people with sarcomas became our main support. Reading about others’ experiences, learning about treatments and promising research broke our isolation and empowered us. Even if only through the internet, my mother was able to relate her experience with that of someone else with the same condition, in the same way as people with hypertension talk about their medication while waiting to see the doctor. Technology is available for any patient, irrespective of the incidence of their condition. It is a double-edged sword: it can provide good quality, evidence based information and totally unreliable, even dangerous information. For patients like my mother, the line between good and bad is hard to draw. Technology should never replace good information and support from trained healthcare professionals.

*“Clinical trials have insufficient numbers of patients to draw meaningful conclusions”*

I remember looking up the Italian guidelines on leiomyosarcoma management. I had just spent weeks learning the Scottish equivalents for common diseases for my exams: management of an acute MI, therapy adjustment for asthma. I naively expected the same level of detail for my mother’s disease. The guidelines can be broadly paraphrased as: “We do not know which chemotherapy works best for uterine sarcomas. Here are a list of drugs you should try in any order.” Given the paucity of evidence and recommendations, I could not blame the oncologist for his reticence to initiate chemotherapy. Equally, I do not blame him for being unaware of the existence of the final chemotherapy agent she received. After all, when my father found about this drug, he agreed to read up on it. He was of further assistance when it became apparent that this drug was not immediately available for people like my mother on the Italian national health service. He made enquiries and arranged its delivery to the local hospital, although the process took three months. Three months my mum did not have.

My mother’s chemotherapy battle highlights a strange paradox only glimpsed by people with rare diseases - patients and their families are often more knowledgeable than the professionals. Doctors are unaware of potentially effective treatments simply because they have never had cause to use them before, or because the drugs are not licensed for a rare disease.

*If I developed a rare disease…*

If I develop a rare condition at the same age my mother was diagnosed, it would be the year 2050. I hope that by then, a family with a relative affected by a rare condition will not have to envy those of people affected by common conditions. I hope that guidelines will be published by professional bodies for a greater number of rare diseases. I hope there will be more multi-centred, multi-national randomised controlled trials and greater resources for treatment, both in terms of drugs and skilled professionals. I hope they will be readily accessible temporally and geographically. Most of all, I hope that nobody ever feels that same sense of fear I felt the first time I realised my mother’s cancer was rare.

## Consent

Written informed consent was obtained from my Father as my Mother’s next of kin for publication of this Letter. A copy of the written consent is available for review by the Editor of this journal.

## References

[1] D’Angelo E, Prat J. Uterine sarcomas: a review. Gynaecol Oncol. 2009; doi:10.1016/j.ygyno.2009.09.023. 10.1016/j.ygyno.2009.09.023 19853898

[2] Cancer Research UK (2012). Uterine cancer statistics.

[3] Rare Disease UK (2015). About rare diseases.

[4] Limb et al. Experiences of rare diseases: an insight from patients and families. 2010. http://www.raredisease.org.uk/documents/RDUK-Family-Report.pdf. Accessed 11 Nov 2015.

[5] Marini F, Facchetti A, Del Monte F, Carbonell-Sala S, Tognarini I, Luzi E, Brandi ML. Multiple endocrine neoplasia type 2. Orphanet J Rare Dis. 2006; doi:10.1186/1750-1172-1-45. 10.1186/1750-1172-1-38 PMC1594566 17014705

